# Intelligent selection of healthcare supply chain mode – an applied research based on artificial intelligence

**DOI:** 10.3389/fpubh.2023.1310016

**Published:** 2023-12-11

**Authors:** Ping Long, Lin Lu, Qianlan Chen, Yifan Chen, Chaoling Li, Xiaochun Luo

**Affiliations:** ^1^School of Economics and Management, Guangxi Normal University, Guilin, China; ^2^Adam Smith Business School, University of Glasgow, Scotland, United Kingdom; ^3^School of Economics and Management, Nanjing University of Aeronautics and Astronautics, Nanjing, China

**Keywords:** healthcare supply chain, mode selection, artificial intelligence, intelligent selection, deep reinforcement learning algorithms

## Abstract

**Introduction:**

Due to the inefficiency and high cost of the current healthcare supply chain mode, in order to adapt to the great changes in the global economy and public health, it is urgent to choose an effective mode for sustainable development of healthcare supply chain. The aim of this paper is to use artificial intelligence systems to make intelligent decisions for healthcare supply chain mode selection.

**Methods:**

Firstly, according to the economic benefits, social benefits and environmental benefits of healthcare supply chain, this paper identifies different healthcare supply chain modes in combination with artificial intelligence technology. Secondly, this paper presents the intelligent choice optimization method of healthcare supply chain mode based on deep reinforcement learning algorithm. Finally, the effect of artificial intelligence in healthcare supply chain mode selection is verified by simulation experiment.

**Results and Discussion:**

The experimental results show that healthcare supply chain mode selected by artificial intelligence is basically consistent with the target mode, while healthcare supply chain mode selected by the basic selection method, BP neural network method and big data method is different from the target mode, which indicates that AI has more advantages in the selection of medical supply chain mode. Therefore, we recommend the application of artificial intelligence to healthcare supply chain management. This study not only makes up for the ineffective problems of existing methods, but also makes up for the gaps in the application of AI technology in the field of healthcare supply chain. The scientific value of this paper is that the proposed framework and the artificial intelligence algorithm enrich the relevant theories of healthcare supply chain research and provide methodological guidance for intelligent decision-making of healthcare supply chain. At the same time, for medical enterprises, this research provides a new practical guideline for the application of artificial intelligence in the sustainable development and modern management of healthcare supply chain.

## 1 Introduction

In the post epidemic era, the industry environment and consumer demand have changed significantly, which has accelerated the digitalization of the healthcare supply chain (1, 2), the development of the modern healthcare supply chain has entered a new era, and the study of the healthcare supply chain has become a hot topic in both academia and industry. The healthcare supply chain plays a crucial role in health economics, as it directly impacts the cost, accessibility, and quality of healthcare services. Specifically, the healthcare supply chain occupies the core position in ensuring the availability and availability of pharmaceutical products and services (3). Studying the healthcare supply chain helps to ensure high-quality healthcare products and services (4) and minimize the risk of harm to patients. Second, studying the healthcare supply chain can help to improve its operational efficiency by minimizing waste, reducing costs, and optimizing resource utilization (5), this can promote the improvement of medical economic level of medical enterprises. Third, during emergencies or epidemics, the healthcare supply chain plays a key role in ensuring the availability of critical supplies (e.g., medicines, personal protective equipment, and medical devices) (6), and a resilient healthcare supply chain can respond quickly to emergencies and minimize losses due to risks. Finally, the healthcare supply chain involves information, logistics, and financial flows from suppliers to healthcare providers and ultimately to patients, which involves multiple stakeholders, including manufacturers, distributors, healthcare providers, and regulatory agencies (7). The research of the healthcare supply chain is beneficial for coordinating the interests of the supply chain actors and enhancing the competitiveness of the supply chain. Therefore, considering the importance of healthcare supply chain in ensuring timely supply of medical products and services, minimizing the risk of patient harm and minimizing waste, as well as the dominant position of healthcare supply chain mode in the supply chain operation process, this paper focuses on the intelligent selection of healthcare supply chain mode.

With the emergence of enterprise clusters, the competition mode among enterprises has begun to transition from single-enterprise competition to supply chain competition, and the competitiveness of supply chains increasingly depends on whether they can meet the requirements of sustainable development (8). The development and pattern evolution of the manufacturing industry have an important impact on the world economy (9), but at the same time, due to the production characteristics of the industry, it also becomes the biggest hidden danger to the ecological environment. The special supply chain such as healthcare supply chain involves a large number of chemical reactions in the process of research and development of pharmaceuticals, which will produce a large number of harmful pollutants such as waste water, waste gas, experimental waste liquid and other harmful pollutants that have a negative impact on the environment. The ingredients contained in these pollutants are also more complex, and if they are not disposed of in a compliant way, it will have a great negative impact on the environment. In addition, the business system of healthcare supply chain covers pharmaceutical research and development, distribution and other fields, and involves multiple links such as procurement, production, delivery, marketing and recycling in daily operation (10). Whether each link meets the “green standard” is closely related to human health. Therefore, some pharmaceutical companies have been implementing environmental protection actions through green practices such as recycling and medical waste treatment, and adopting new clean technologies such as low-pollution gas heating/cooling, electric vehicles and energy-saving policies to increase social benefits (11). Therefore, in order to meet the new industry needs, there is a need to study the optimal mode of healthcare supply chain and use modern decision-making technology tools.

At the same time, the application of AI appears in a number of different fields, including the supply chain (12). AI systems are able to make informed decisions and perform tasks automatically without human intervention. The implantation of AI systems in the supply chain is still an innovation, and AI is expected to optimize and upgrade various industries, including medical enterprises (13). As far as the healthcare supply chain is concerned, AI offers plenty of opportunities to improve operational efficiency and sustainability. Specifically, firstly, artificial intelligence algorithms help healthcare supply chain to forecast demand and manage inventory (14). Artificial intelligence can minimize waste, improve cost efficiency, and ensure timely supply of key materials. Secondly, artificial intelligence algorithms can optimize the logistics of healthcare supply chain, improve transportation efficiency, reduce fuel consumption, and minimize carbon emissions, thus promoting the sustainable development of healthcare supply chain. Finally, artificial intelligence has significant advantages in continuous improvement and learning. It can clearly and quickly identify the optimal operation mode of healthcare supply chain through the analysis of big data (15), which makes healthcare supply chain more flexible. To sum up, artificial intelligence plays a vital role in promoting the development of health economy, and the integration of artificial intelligence technology into all aspects of healthcare supply chain operation can bring significant benefits (16). This provides technical support for the healthcare supply chain to gain competitive advantages, improve operational efficiency, and become a more low-carbon and environmentally friendly medical system with sustainable development potential (17).

However, few scholars have conducted in-depth studies on the issue of healthcare supply chain mode selection. At the same time, given the great advantages of AI technology in optimizing healthcare supply chain, there are few researches on the role of AI technology in healthcare supply chain mode selection. Therefore, to fill this gap and given that deep reinforcement learning algorithms are particularly suitable for highly variable environments and applicable to the research scenarios of the article, we propose a deep reinforcement learning algorithm based on AI with high scalability to optimize healthcare supply chain mode selection. Since AI technology has a crucial role in the development of healthcare supply chains and is valuable for optimizing healthcare supply chain modes and improving competitiveness, this is the main motivation for this study. In addition, the competition of current business modes is becoming more and more intense, and enterprises need to find suitable modes to realize their sustainable development, which further illustrates the necessity of applying AI technology to the selection of healthcare supply chain modes. In summary, the article identifies the following three research objectives:

(1) Determine the indicators for evaluating healthcare supply chain modes and clarify the relevant calculation methods to support the effective selection of the optimal healthcare supply chain mode;
(2) Combined with the evaluation indexes, different healthcare supply chain modes are identified using artificial intelligence technology, and an intelligent selection optimization method for healthcare supply chain modes is proposed based on deep reinforcement learning algorithm;
(3) Constructing simulation experiments to verify the effectiveness of the application of artificial intelligence technology.

This study has three major contributions to the field of healthcare supply chain research. First, we discuss a neglected and understudied problem in previous studies: how to choose the optimal healthcare supply chain mode? Based on the characteristics of healthcare supply chain, this paper uses artificial intelligence technology to choose healthcare supply chain mode, and puts forward a new method of healthcare supply chain mode selection, which enhances the objectivity and effectiveness of the selection process. Secondly, based on the deep reinforcement learning algorithm, this paper puts forward a specific algorithm to optimize the mode selection of healthcare supply chain, enriching the relevant theories and methods of healthcare supply chain research, and ensuring the feasibility of the results. Finally, the simulation experiment verifies the effectiveness of artificial intelligence in the application of healthcare supply chain mode selection, which provides new practical guidance and valuable insights for artificial intelligence to improve the level of health economy, sustainable development of medical supply chain and modern management.

The paper is organized as follows. Section 2 will review the literature on research on healthcare supply chain and AI applications. Section 3 describes the related research methodology and the process of intelligent selection of healthcare supply chain modes. Section 4 conducts simulation experiments for validation. Section 5 is a discussion and analysis of the results of the simulation experiments. Section 6 is the conclusion and further indicates the future research direction.

## 2 Literature review

### 2.1 Healthcare supply chain mode selection

Existing research on healthcare supply chain is very rich and many scholars have explored different aspects of healthcare supply chain in depth. For the logistics process of healthcare supply chain, Smith et al. argued that the logistics process of healthcare supply chain has become a key factor affecting the cost and quality of healthcare services, and they conducted two surveys of healthcare supply chain professional practitioners to understand the operational efficiency of the healthcare supply chain and the potential opportunities for improvement, and the article pointed out the benefits and difficulties of standardizing data in the healthcare supply chain, as well as how managers can provide support in achieving data standardization (18). Addressing the aspect of healthcare supply chain digitization and performance, it was noted that the global manufacturing industry is working hard to improve productivity by leveraging digital manufacturing technologies such as artificial intelligence and the Internet of Things (IoT), which are expected to improve the connectivity between supply chains, and this trend extends to the healthcare supply chain sector. Meanwhile, social capital, a potential resource for healthcare supply chains, can affect supply chain performance in the healthcare industry. Therefore, the article conducted an empirical study on the relationship between healthcare supply chain digitization, social capital and supply chain performance (19). For risk management in healthcare supply chain, some studies have made guidance for improving healthcare supply chain practices by revealing the relationship between supply chain integration, supply chain risk management, and supply chain 4.0 and healthcare supply chain performance, so that healthcare supply chain can better cope with risks and enhance performance (20).

It is not difficult to see that many scholars have made certain achievements in the study of healthcare supply chain, and most of the research content is closely related to the operation of healthcare supply chain, based on which, it is necessary to mention the healthcare supply chain mode, which dominates the various stages of the supply chain, and largely determines the structure of the healthcare supply chain and its operation mode. The choice of healthcare supply chain mode plays a fundamental role in the development of the supply chain, specifically, from the viewpoint of patient safety, an effective healthcare supply chain mode can help control the quality and safety of medicines (21), and filter the potential risks and loopholes that may affect the safety of patients to ensure the safety of patients. From the perspective of cost control, an appropriate healthcare supply chain mode is conducive to improving inventory management, streamlining processes, and optimizing logistics as a means to achieve cost savings. From the perspective of healthcare supply chain risk management, an efficient healthcare supply chain operation mode is conducive to making quick and effective decisions to reduce risks and ensure the continuity of healthcare supply chain operations. From the perspective of sustainable development, an excellent good healthcare supply chain mode is conducive to the reduction of carbon emissions, which improves economic benefits and ensures the growth of environmental and social benefits at the same time.

However, there is little literature studying the selection of healthcare supply chain modes. In terms of the existing researched healthcare supply chain modes, one study proposed the healthcare supply chain e-commerce mode (22), which is the combination of e-commerce and healthcare supply chain. This mode solves the drawbacks existing in the traditional healthcare supply chain, optimizes the structure of the healthcare supply chain, and improves the efficiency of the healthcare supply chain. There is also a study on the SPD mode of the healthcare supply chain (23), which refers to a medical supplies management mode integrating supply/processing/distribution links, and its core carrier is a medical consumables supply chain platform, aiming to help medical institutions realize refined operation and cost control. The SPD mode comprehensively takes into consideration the operation rules and characteristics of each management link of medical consumables in hospitals, as well as the interconnection between links. With the support of supply chain management theory and information technology, it optimizes and improves the traditional way of medical consumables management, and is a consumables management mode applicable to the current social and medical background.

In terms of the methodology of the healthcare supply chain mode selection, some scholars created a vaccine supply chain model in response to the global crisis and used a multi-objective mixed integer planning (MIP) model to develop the supply chain (24), and this study also integrated the TOPSIS (Order Preference Technique by Similarity to Ideal Solution) model into the optimization model to identify the best solution from a set of non-dominated solutions that prioritize environmental sustainability, although the algorithm used in this study can be used to design a sustainable vaccine supply chain mode, it has a narrow scope of application and the study can be expanded to utilize other hybrid meta-heuristic algorithms for the analysis of the experimental results for the purpose of comparing the findings. Other scholars have finally constructed and proposed a new model of a sustainable healthcare supply chain that prioritizes patient safety by identifying key attributes of a sustainable healthcare supply chain and then applying the Fuzzy Delphi Method (FDM) to determine the appropriateness of these key attributes and categorize them in order to finally construct and propose a new mode of a sustainable healthcare supply chain that prioritizes patient safety, which improves the safety of healthcare professionals and patients in a sustainable manner (25). Furthermore, to deal with the risks faced in the operation of the supply chain. Rajak and other scholars used the hybrid BWM-QFD methodology to identify the stakeholders’ requirements and critical success factors (CSFs) for the sustainability initiative in SC during this pandemic situation (26). Because the choice of healthcare supply chain mode needs to focus on the impact on the sustainable development of medical supply chain. The framework proposed by the study and the 16 key success factors identified provide good solutions to supply chain sustainability problems. Thus, about the measurement of sustainability dimensions in healthcare supply chain mode selection, this study provides important methodological references and content guidance. Finally, an integrated DEA enhanced Russell measure (ERM) model was studied to select the best transportation system provider. This model is used for the performance evaluation of the transportation system to minimize capital cost, cost of energy consumption, cost of work safety and labor’s health, and CO_2_ emission (27). In terms of healthcare supply chain mode selection, the assessment of medical goods transportation, capital costs, energy consumption, exhaust emission and other indicators are particularly important. The value obtained by these indicators will directly affect the choice of healthcare supply chain mode. Therefore, this model provides strong method support for the selection of healthcare supply chain transportation system, which is important for the selection of effective healthcare supply chain mode.

### 2.2 Application of artificial intelligence in the healthcare supply chain

With the rapid development of a series of emerging technologies such as big data and cloud computing, artificial intelligence technology is also increasingly used in the medical field (28). As an advanced technology, artificial intelligence can use the high-speed processing and distributed computing ability of computers to simulate the information processing ability and learning ability of the human brain, so that computers can analyze and solve certain problems that cannot be handled by medical technology (29), and the feasibility of the application of AI technology in the field of healthcare has been confirmed, and the future prospects are broad (30).

Currently, the application of artificial intelligence technology in the healthcare supply chain is mainly reflected in the following five aspects. First, the application of artificial intelligence technology in pharmaceutical research and development (31), the significant changes in the field of pharmaceutical research and development are closely related to the breakthrough progress in the field of artificial intelligence (32). In the face of the extremely large pharmaceutical industry data, AI can be used to process the data through appropriate algorithmic models, and then used in the process of pharmaceutical R&D, which can effectively accelerate the speed of pharmaceutical product development, reduce the cost of R&D, and improve the success rate of research and development. Second, AI can improve the sustainability of the healthcare supply chain (33). Obviously, AI technology has gradually become a strategic tool to improve competitiveness, and the application of AI in all aspects of the supply chain guarantees the normal operation of the business, and its fast and effective decision-making and execution ability prevents the disruption of the healthcare supply chain after the impact. Third, AI technology can be used in healthcare supply chain management (34). The healthcare supply chain is a very complex network, and the use of innovative tools for healthcare supply chain management can create economic surpluses and take into account the interests of the supply chain actors. Fourth, AI techniques can be used for demand forecasting and patient health management (35). Artificial intelligence analyzes and compares the patient’s indicators with the standard data by using the data analysis system and transmits them to the server’s terminal, and finally gives reasonable suggestions. This technology can combine relevant data to assess personal health, establish personal health service files, and provide health control for key patients. Fifth, artificial intelligence technology can be used to assist in the diagnosis of medical imaging (36) and improve the efficiency of the healthcare supply chain. Traditional medical diagnosis is mainly obtained by relying on the doctor’s analysis of the image data, mostly by virtue of personal experience as well as subjective judgment, and therefore the phenomenon of misdiagnosis will occur. Medical imaging can be divided into two parts: imaging and image analysis, and the use of artificial intelligence technology can make imaging faster, effectively shorten the imaging time and improve the clarity of the image. In the image analysis part, artificial intelligence technology can quickly extract data from the image database and analyze it to help doctors carry out auxiliary diagnosis, avoiding diagnostic errors due to doctors’ subjective factors, which effectively improves the accuracy of diagnosis.

However, the application of AI in the healthcare supply chain field still has some challenges and limitations. First of all, the application scope of artificial intelligence in the field of healthcare supply chain is relatively narrow (37). Compared with other fields (such as retail industry and banking industry), the healthcare supply chain is subject to more strict legal constraints, and its data security and privacy protection are more important, which limits the application of artificial intelligence in the medical field to a certain extent. The research of artificial intelligence in helping the healthcare supply chain make intelligent decisions needs to be further explored. Second, the public health system is gradually transforming, its main focus is to realize the value chain through the use of digital technology (38), while past research has mainly discussed the scalability of artificial intelligence and some ethical issues, as well as how to realize the value chain in the medical field in the new business environment has not been clarified. More theoretical and empirical research is needed to address the challenges facing AI. Finally, the current research cognition of artificial intelligence is still limited and scattered, which may be caused by the disjoint of previous research. Some scholars only focus on the application of AI in disease monitoring and drug development (39, 40), and lack a comprehensive understanding of artificial intelligence systems, which limits the practical value of artificial intelligence. In fact, the application of artificial intelligence can benefit different subjects in the healthcare supply chain (41).

In short, with the development of the times, artificial intelligence technology has been serving more and more aspects of the healthcare supply chain. The popularity of artificial intelligence technology has greatly improved the degree of intelligent development of the medical system. However, in view of the limitations and challenges existing in the application of artificial intelligence in the field of public health, the application of artificial intelligence in the choice of healthcare supply chain mode is still a blank. Further discussion is needed.

### 2.3 Research gaps

To sum up, it is not difficult to see that the existing research literature on healthcare supply chain and artificial intelligence is relatively rich and the research system is relatively mature. Most scholars focus on discussing the application of artificial intelligence in healthcare supply chain from the perspectives of sustainable development, supply chain management and logistics, so they ignore the research on healthcare supply chain mode. In particular, there are few researches on the application of artificial intelligence in the selection of healthcare supply chain modes. In addition, in view of the existing healthcare supply chain mode selection methods, the significant problem is the lack of systematicness and objectivity, and it is difficult to apply such methods in practical analysis.

Healthcare supply chain mode determines the structure and operation mode of the supply chain to a large extent. Only by choosing an effective mode can healthcare supply chain have an advantageous competitive position in the highly competitive market. And it is conducive to the long-term development of the supply chain. The selection of healthcare supply chain mode needs to be interpreted from a deeper perspective, and the existing research methods and tools need to be improved and optimized. Therefore, this paper not only makes up for the gaps in the existing healthcare supply chain research content, but also makes up for the defects of research methods.

## 3 Research methodology

### 3.1 Deep reinforcement learning algorithm

Reinforcement Learning (RL), also known as evaluation learning, is an important branch of machine learning. Its feature is that there is no prior knowledge, it does not need to give the correct action, but only needs to give the return, and adjust the strategy to maximize the total cumulative return. To put it simply, reinforcement learning is that the actions generated by an Agent interact with the environment and generate sample sequences. Through continuous learning, the agent searches for actions with high returns and avoids actions with low returns. Obviously, reinforcement learning is a trial-and-error process that simulates the learning process of animals. It obtains reward and punishment information by constantly trying actions and iteratively updates its reward value, so as to find the optimal solution. Since reinforcement learning is the reward information obtained from environmental feedback in the learning process, reinforcement learning is applicable to unknown environments and has decision-making ability (42). The paradigm of reinforcement learning is very similar to the process of human learning knowledge, so reinforcement learning is regarded as an important way to achieve general AI (43), but it is helpless to solve the perception problem.

Deep learning is one of the most popular branches of machine learning in recent years, which has made great achievements in image recognition and natural language processing. The simple explanation of deep learning is that when we know an input and mapping, we can get its output, but the reality is that the mapping is very complex, cannot be expressed in an expression or simply unknown state. At this time, deep learning constructs a deep neural network as the connection bridge between sample input and feature output. We constantly input samples for training, so that the neural network can fit and approximate a mapping corresponding to these samples as much as possible, and the finally trained network can be approximately equivalent to the mapping we need. Although deep learning has strong perception ability, it lacks certain decision-making ability.

Therefore, in recent years, with the continuous improvement of deep learning theory, the combination of reinforcement learning and deep learning has received more and more attention and research, and the deep reinforcement learning algorithm has gradually become a research hotspot in the field of artificial intelligence (44). Introducing deep learning into the reinforcement learning framework and replacing the original Q-table with neural network can greatly improve the computing power of the reinforcement learning algorithm and deal with higher dimensional and more complex situations. In other words, the neural network provides the perception ability for reinforcement learning that it lacks. By combining the fitting approximation ability of deep learning with the strategy generation ability of reinforcement learning, the transformation from table form to network form is achieved, and deep reinforcement learning is formed. Choosing deep reinforcement learning algorithm for healthcare supply chain mode selection mainly takes into account the unique functions and characteristics of deep reinforcement learning. First of all, the healthcare supply chain mode selection involves a complex decision-making process, which requires the AI model to learn in a dynamic and uncertain environment. Among many AI algorithms, DRL is the most suitable for unknown environment and has strong decision-making ability. Second, in healthcare supply chain mode selection, decisions made at one stage may affect subsequent stages, DRL’s ability to learn from sequential interactions enables it to effectively address such impacts. Finally, healthcare supply chain mode selection needs to balance multiple objectives, such as economic cost, patient safety and environmental sustainability. DRL can learn more complex decision spaces through interaction with the environment and identify solutions that are consistent with the organization’s goals to achieve a balance between conflicting goals. In short, the key to choosing the deep reinforcement learning model over other artificial intelligence models for healthcare supply chain mode selection is that, compared with other artificial intelligence algorithms, DRL is more suitable for uncertain environments and can handle complex and continuous decision-making tasks. These characteristics make DRL very suitable for solving the multi-faceted challenges of healthcare supply chain management.

**Figure 1:**
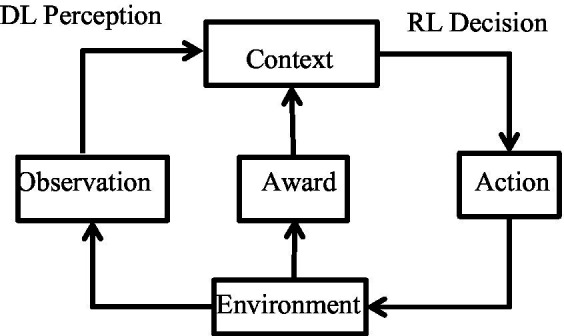
DRL schematic framework.

Current deep reinforcement learning algorithms mainly include two types, namely deep reinforcement learning algorithms based on value function approximation and deep reinforcement learning algorithms based on policy gradient. Among them, the most representative ones in the field of value function approximation algorithms are Deep Q network algorithm (DQN) (45) and dual Deep Q network algorithm (DDQN) (46). The most representative in the field of policy gradient algorithms is the depth deterministic policy gradient algorithm (DDPG) (47). In deep reinforcement learning, we use neural networks to represent value functions and strategies and solve their modeling problems. We continuously input samples to continuously improve the values of weight W and bias b in the network, and use Error Back Propagation (BP) methods to continuously iterate and optimize our objective functions. The network can approximate the real value function and strategy as much as possible. The DRL framework is shown in Figure 1, and its learning process can be described as follows:

(1) At each moment agent interacts with the environment to get a high-dimensional observation, and utilizes the DL method to perceive the observation to get a specific state feature representation.
(2) Evaluate the value function of each action based on the expected return and map the current state to the corresponding action by some strategy.
(3) The environment reacts to the action and obtains the next observation. By continuously cycling the above process, the optimal strategy to realize the goal can be obtained eventually.

### 3.2 Design of intelligent selection method of healthcare supply chain mode

**Figure 2:**
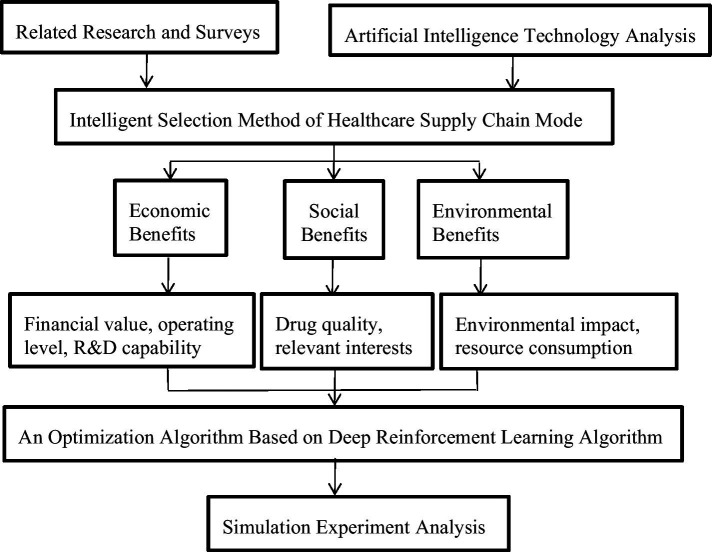
Intelligent selection process for healthcare supply chain mode.

In view of the current healthcare supply chain mode selection problem, there is no specific method to help healthcare institutions choose the optimal mode, and the current research ignores the potential of technology and innovation in optimizing the healthcare supply chain. Therefore, this study applied artificial intelligence technology to the intelligent choice of healthcare supply chain mode, designed a new intelligent choice method and adopted a more scientific calculation method to select the optimal healthcare supply chain mode to achieve sustainable development. In this study, the intelligent selection process of healthcare supply chain mode is set up as shown in Figure 2. This study mainly provides the basis for the selection of healthcare supply chain mode through indicators such as economic benefit, social benefit (48) and environmental benefit (49). By considering the economic, social, and environmental dimensions of each supply chain mode, healthcare companies can make informed decisions to optimize their supply chain strategies while promoting sustainability, ethical practices, and cost-effective operations. The selection process involves a lot of calculation parts, in order to ensure the scientific selection results, it is necessary to ensure the feasibility of the calculation algorithm.

#### 3.2.1 Economic benefits of healthcare supply chain

The economic efficiency reflects the overall economic operation effect of the healthcare supply chain, which is the performance of the ability of capital circulation. The economic benefits of healthcare supply chain include financial value, internal operation and management level of the supply chain, and pharmaceutical R&D innovation capability.

For the financial value of the healthcare supply chain, this article selects the total asset turnover rate, the profit rate of pharmaceutical sales and the current ratio of the healthcare supply chain as the indicators for the assessment of the financial value, which fully reflects the financial operation status and profitability of the entire healthcare supply chain.

(1) Total Asset Turnover Ratio. This index mainly measures the ratio between the net income from pharmaceutical sales and the asset investment scale of healthcare supply chain in a certain period of time, which to a certain extent can reflect the operational efficiency of total assets of healthcare supply chain. The larger the value of this index, the higher the level of sales of medicines in the healthcare supply chain, the greater the return on asset investment, and the stronger the operating ability of the total assets of the supply chain.

(1) $$\mathrm{Total asset turnover}=\frac{\begin{array}{l} \mathrm{Net}\;\mathrm{proceeds from the}\\ \mathrm{sale of pharmaceuticals} \end{array}}{\begin{array}{l} \mathrm{Total healthcare}\\ \mathrm{supply chain assets} \end{array}}\times 100\%$$

(2) Profitability of pharmaceutical sales. This indicator is the ratio of total profit to product sales revenue. It reflects the profitability of the product and indirectly reflects the size of the competitiveness of the enterprise’s products in the market.

(2) $$\mathrm{Profitability of pharmaceutical sales}=\frac{\mathrm{Sales profit}}{\mathrm{Total sales}}\times 100\%$$

(3) Current ratio. The current ratio is the ratio of current assets to current liabilities of the healthcare supply chain for a certain period of time. The current ratio can reflect whether the healthcare supply chain has enough working capital for production to supply products to the demand side.

(3) $$C\mathrm{urrent ratio}=\frac{\mathrm{Current asset}}{\mathrm{Current liabilities}}\times 100\%$$

The healthcare supply chain involves many aspects such as procurement, production, logistics, sales, and reverse logistics. It is necessary to ensure the overall circulation of the healthcare supply chain as a whole in all links. Therefore, each link is crucial. Based on the characteristics of the healthcare supply chain and the acceptability of the indicator data, the article selects the two indicators of pharmaceutical inventory turnover rate and the healthcare supply chain management cost rates to evaluate the internal operation and management level of the healthcare supply chain.

(1) Pharmaceutical inventory turnover rate. Inventory turnover rate is an important indicator for measuring the management capacity of healthcare supply chain inventory. The higher the value of this indicator, the faster the represented inventory transition to the capital.

(4) $$\begin{array}{c} \mathrm{Pharmaceutical inventory}\\ \mathrm{turnover rate} \end{array}=\frac{\mathrm{Sales cost}}{\mathrm{Average inventory balance}}\times 100\%$$

(2) The healthcare supply chain management cost rate. Management cost rate can reflect the current cost of the current healthcare supply chain operation cost.

(5) $$\mathrm{Management cost rate}=\frac{\mathrm{Total operating cost}}{\mathrm{Total operating income}}\times 100\%$$

Pharmaceutical research and development and innovation capabilities are the key factors affecting the sustainable development of the healthcare supply chain. Only when healthcare supply chain continues to launch new products and services can the supply chain be maintained and effectively expand its market share. The healthcare supply chain pharmaceutical R&D and innovation capabilities are measured by two indicators of new technologies and research and development investment rates.

(1) New technology adoption rate. The adoption rate of new technology refers to the ratio of the output value of the new technology product to the total output value of the enterprise. This indicator reflects the contribution of new technologies to the total value of corporate products.

(6) $$\mathrm{New}\;\mathrm{technology adoption rate}=\frac{\begin{array}{l} \mathrm{Gross}\;\mathrm{new}\;\mathrm{technology}\\ \mathrm{product value} \end{array}}{\mathrm{Gross product value}}\times 100\%$$

(2) R&D investment rate. This index refers to the proportion of the cost of healthcare supply chain for research and development over a certain period of time in the total operating income of the supply chain, reflecting the importance of healthcare supply chain to innovation development.

(7) $$R\&D\;\mathrm{investment rate}=\frac{R\&D\;\mathrm{total investment}}{\mathrm{Total operating income}}\times 100\%$$

#### 3.2.2 Social benefits of healthcare supply chain

The supply of high-quality drugs is one of the main targets of healthcare supply chain operations, and the quality of drugs must have strict implementation standards. The quality of drugs can be measured from the three indicators of pharmaceutical qualification rate, pharmaceutical return rate, and market share.

(1) Pharmaceutical qualification rate. The qualified rate of drugs refers to the percentage of the number of qualified drugs in a certain period of time. For a certain period of time, the medical supply chain production drug A, a total of N batches. The output of the i(1≤i≤N) is T , of which the number of qualified products is Qi ,the qualification rate of the drug during this period is:

(8) $$RQA=\frac{\overset{N}{\underset{i=1}{\sum }}Qi}{\overset{N}{\underset{i=1}{\sum }}Ti}\times 100\%$$

(2) Pharmaceutical return rate. Product return rate refers to the percentage of the number of drugs in the total supply of drugs in a certain period of time. For a certain period of time, the healthcare supply chain issues a total of N batches for drug A. The supply volume of the i(1≤i≤N) is Si . The number of returns is Ri . The return rate of the drug product during this period is:

(9) $$RRA=\frac{\overset{N}{\underset{i=1}{\sum }}Ri}{\overset{N}{\underset{i=1}{\sum }}Si}\times 100\%$$

(3) Market share. The market share of drugs is a key indicator of measurement of the competitiveness of the drug market. It can be expressed as the ratio of drugs in a regional medical supply chain to account for the proportion of similar drugs in the region. For the sales of drugs supplied by a certain period of healthcare supply chain is $S^{\prime }$ , the total number of pharmaceutical sales in the region is S _,_the market share of pharmaceutical is:

(10) $$R\mathrm{MS}=\frac{S^{\prime }}{S}\times 100\%$$

During the management of healthcare supply chain, enterprises need to regularly conduct education and training of employees to help employees improve their learning ability. This is not only the manifestation of the responsibility of enterprises in terms of employee management, but also the obligation of the enterprise. Therefore, the growth rate of the total number of employee training in this paper reflects the protection of employees’ rights and interests of pharmaceutical manufacturing enterprises. In a certain period of time, the total number of employee training changes from NT to the end of $NT^{'}$ , and the total growth rate of employee training is:


(11) $$RT=\frac{NT^{'}-NT}{NT}\times 100\%$$

The embodiment of shareholders’ equity is mainly due to the legitimate rights of shareholders. All business behaviors must be carried out on the premise that the capital security of shareholders can ensure the security of shareholders and whether it can be divorced and creating profits on time. The article uses two indicators of net assets per share and net assets to measure shareholders’ rights in the medical supply chain.

(1) Net assets per share. This indicator measures the number of assets that shareholders can obtain in each shareholder issued by an enterprise.

(12) $$\mathrm{Net}\;\mathrm{assets}\;\mathrm{per}\;\mathrm{share}=\frac{\mathrm{Total shareholder equity}}{\mathrm{Total shareholders}}\times 100\%$$

(2) Net asset yield. This indicator refers to the percentage share of net profit obtained by medical supply chain sales during a period of time.

(13) $$\mathrm{Net}\;\mathrm{asset yield}=\frac{\mathrm{Total profit of drug sales}}{{\mathrm{Owners}}^{'}\;\mathrm{equity}}\times 100\%$$

In the process of medical supply chain management, it is also important to protect the rights and interests of creditors. For creditors, the most concerned question is whether the enterprise has the ability to pay debt. Therefore, the article selects the asset -liability ratio to measure the ability of the medical supply chain.

(1) Asset-liability ratio. Asset-liability ratio refers to the ratio of the total liabilities of the healthcare supply chain in a certain period of time. This indicator is a comprehensive indicator of evaluating the level of healthcare supply chain liabilities, and an important criterion for measuring its degree of risk.

(14) $$\mathrm{Asset - liability ratio =}\frac{\mathrm{Total liability}}{\mathrm{Total asset}}\mathrm{\times 100}$$

#### 3.2.3 Environmental benefits of healthcare supply chains

The environmental benefits of healthcare supply chain is an important index to highlight whether the supply chain is “green,” measuring the environmental benefits of healthcare supply chain meets the requirements of the present era. This paper evaluates the environmental impact, resource consumption intensity and ISO certification rate of healthcare supply chain.

(1) Environmental impact of healthcare supply chain. Total waste emissions from healthcare supply chain. This index refers to the sum of three types of waste emissions from a series of supply chain activities, such as production by pharmaceutical manufacturing enterprises.
(2) Resource consumption intensity. The type, quantity and scarcity of medical resources used in the operation of the pharmaceutical supply chain.
(3) ISO certification rate. The enterprise through the implementation of a series of standards, the implementation of quality system certification, proof of its technical ability to meet customer requirements, provide the right products. The number of ISO certifications achieved in a medical supply chain is NISO , and the number of ISO certifications required in the industry is NNISO , the company’s ISO certification rate is:

(15) $$RISO=\frac{NISO}{NNISO}\times 100\%$$

### 3.3 Optimization algorithm based on deep reinforcement learning

Based on the above indicators and related computing formulas, with the help of the artificial intelligence system, the selection of the healthcare supply chain mode in this section is completed. This paper has selected three healthcare supply chain modes through artificial intelligence technology.

#### 3.3.1 Healthcare supply chain cooperative mode

In this mode, medical institutions cooperate with pharmaceutical logistics enterprises or supply chain service providers, and pharmaceutical logistics enterprises participate in pharmaceutical inventory and management. Medical institutions have the choice of suppliers, centralized control of consumables, drugs acceptance, warehousing, distribution and other activities to ensure the quality of medical materials. Under the cooperative healthcare supply chain mode, the economic, social and environmental aspects have been greatly improved. In terms of economy, the cooperative mode of healthcare supply chain can achieve economies of scale, reduce procurement costs and achieve total cost savings through collective bargaining, centralized procurement and resource sharing. Secondly, the participants in healthcare supply chain can reduce personal financial risks by sharing the financial burden of procurement, inventory management and logistics. Socially, the collaborative mode of the healthcare supply chain promotes community engagement by supporting local suppliers and businesses, and promotes local economic development and spreads social well-being by prioritizing local procurement. Environmentally, the collaborative mode of the healthcare supply chain supports the development of a green environment by encouraging the use of environmentally friendly products, materials and logistics. Healthcare supply chains under the cooperative mode reduce carbon emissions by consolidating orders, optimizing logistics and sharing distribution networks, and minimize the environmental impact of their supply chain activities. Overall, healthcare supply chain cooperative mode can address economic, social and environmental issues through cost reduction, community engagement and sustainable practices. The main feature of this mode is to open up the capital flow, logistics and information flow, and greatly improve the management level of healthcare supply chain.

#### 3.3.2 SPD mode of healthcare supply chain

SPD mode is also known as the third-party oriented mode. In the SPD mode, the third-party supply chain service provider uses modern information technology to manage the logistics, information flow and capital flow of the supply chain of medical institutions in a unified manner, aiming to build a value-adding, quality and efficiency improvement, and innovative service-oriented healthcare supply chain management system. Economically, effective SPD management can extend the service life of medical devices and equipment and reduce the frequency of replacement. This method minimizes the expenditure of fixed costs. Secondly, SPD supply chain service provider, as a logistics center, provides medical institutions with centralized procurement, warehousing and distribution services of medical consumables, helps medical institutions realize “one-to-one” procurement mode and zero inventory management of materials, realizes effective utilization of resources and reduces warehousing and logistics costs in the operation process. Socially, the SPD mode maintains high standards of sterilization handling and the rational use of safe medical devices, which are essential to ensure patient safety. Second, the SPD mode supports regular training and self-development of employees, ensuring the well-being of employees. Finally, management under the SPD mode protects the interests of creditors. In terms of the environment, the SPD mode reduces waste emissions through the efficient treatment of medical waste. Secondly, through effective sterilization treatment measures, the disposal of disposable items is optimized, contributing to environmental sustainability. The main feature of this mode is to organically integrate the logistics, information flow and capital flow of the supply chain of medical institutions and third-party supply chain service providers into one system, so as to reduce the storage cost of medical institutions and improve the efficiency of material distribution.

#### 3.3.3 Healthcare supply chain e-commerce mode

It consists of healthcare supply chain + e-commerce platforms, and its development goal is to promote the transformation of traditional healthcare supply chain to smart medical supply chain. On the economic side, e-commerce platforms can achieve cost-effectiveness through simplified procurement processes, competitive pricing, and reduced overhead costs. E-commerce platforms facilitate efficient procurement and price negotiations by directly connecting medical institutions with suppliers, contributing to overall cost savings. Second, the healthcare supply chain e-commerce mode improves supply chain transparency, enabling healthcare organizations to make informed purchasing decisions, and this transparency can lead to better cost management and budget allocation. In terms of society, the e-commerce mode improves the accessibility of medical supplies, especially for remote areas or areas with insufficient medical supplies. By providing a digital market for purchasing medical products, people can quickly obtain medical products, so as to realize the social responsibility of enterprises. In terms of the environment, the healthcare supply chain e-commerce mode can maintain environmental protection by reducing the demand for physical transportation and the associated carbon emissions. Second, e-commerce platforms influence consumer preferences and promote the development of green practices by offering environmentally friendly and reusable products. The main feature of this mode is to reduce the hierarchical structure of the healthcare supply chain through Internet technology, enhance the information sharing and communication between various subjects in the medical supply chain, and integrate the Internet into the medical logistics to create an Internet + medicine distribution mode, and improve the operational efficiency of the healthcare supply chain.

This selection is optimized based on the Deep Deterministic Strategy Gradient Algorithm(DDPG) in the reinforcement learning algorithm (50, 51). DDPG algorithm combined with neural network for value function model approximation and deterministic strategy selection. This algorithm requires fewer samples and has high training efficiency. In addition, this algorithm is a reinforcement learning method based on deep learning related theories and technical methods, and takes deterministic strategy gradient as its optimization goal.

DDPG algorithm has four networks:

(1) Actor online network Q : Responsible for the iterative update of parameter θ , while selecting the current action a according to the current state s, interacting with the environment to get the state s' and rewarding r .
(2) Actor target network $Q^{\prime }$ : Select the best action a' from the next state s' , $\theta^{\prime }$ is the parameter.
(3) Critic online network μ : Responsible for the iterative update of parameter ω . Calculate the current value function at the same time, and the expression of the target Q value is as follows:

(16) $$y_{i}=r_{i}+\gamma Q^{'}\left(s^{\prime },a^{\prime },\omega^{\prime }\right)$$

λ represents the discount function.

(4) Critic target network μ' : Calculate $Q^{\prime }$ in the target Q value, $\omega^{\prime }$ is the parameter.

The network parameters in DDPG are updated as shown in equation (17):


(17) $$\{\begin{array}{l} \theta^{\prime }\leftarrow \tau \theta +\left(1-\tau \right)\theta^{\prime }\\ \omega^{\prime }\leftarrow \tau \omega +\left(1-\tau \right)\omega^{\prime } \end{array}$$

τ is the update coefficient, generally selected a small number, usually 0.01 or 0.001.

In the learning process, in order to reduce the regularity of learning, a noise N is added to the selected action a , and the obtained action becomes:


(18) a=πθ(s)+N

Update the Critic network according to Critical loss:


(19) $$L=\frac{1}{N}\overset{N}{\underset{i}{\sum }}{\left(yi-Q\left(si,ai|\omega \right)\right)}^{2}$$

Update the Actor online network:


(20) $$\nabla \theta \mu \mu |\mathrm{si}≅\frac{1}{N}\underset{i}{\sum }\nabla \mathrm{ai}Q\left(\mathrm{si,a}i|\omega \right)\nabla \theta \mu \left(si|\theta \right)$$

Artificial intelligence DDPG algorithm is used to select the medical supply chain model, and its process is mainly as follows:

1. Build a simulation environment.
2. Design a container BUFFER with storage data and random sampling.
3. Build model, define DDPG algorithm, specify loss function and optimizer. The Agent will interact with its environment and learn from it. After the interaction, it will obtain a state’s provided by the environment. After receiving the state, the agent will use the actor network to calculate the action a that should be taken in the current state. Meanwhile, the agent will predict the Q value corresponding to action A in this state according to the critic network. When the action is fed back to the environment, the environment will give the corresponding reward r, the new state’s, and whether to trigger the termination condition done. After each interaction is completed, the DDPG algorithm will store it as an experience in the experience pool and extract a certain amount of experience from the experience pool as input data to train the neural network. Through the execution of multiple rounds of training, constantly adjust the parameters to achieve better results.
4. Evaluate and test the trained model and observe the reward.
5. Save the model to a specified location for subsequent reasoning or continued training.
6. Test the performance of the model in the test set data.

**Figure 3:**
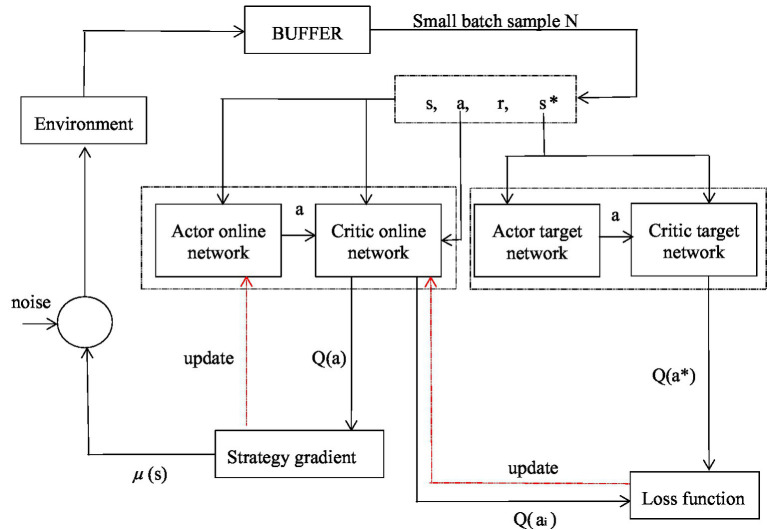
DDPG algorithm framework.

Thus, the healthcare supply chain mode can be determined according to the above algorithm. The overall framework flow chart of DDPG algorithm is shown in the Figure 3. At this point, the intelligent selection method of healthcare supply chain mode based on artificial intelligence technology has been designed and completed.

## 4 Simulation experiment

### 4.1 Simulation experiment plan

**Figure 4:**
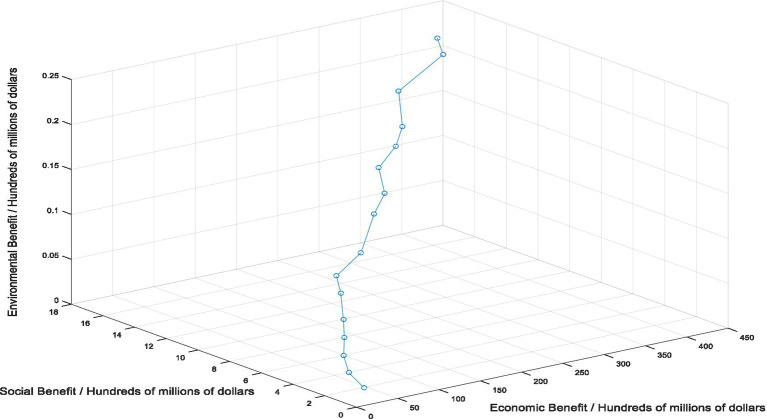
Optimal mode setting for healthcare supply chain.

In this study, in order to make up for the shortcomings of the current healthcare supply chain mode selection method, the intelligent selection method of healthcare supply chain mode based on artificial intelligence technology is proposed. To verify the application effect of this method, constructing simulation experiment to verify the effect of artificial intelligence method. Experimental environment settings: in the DDPG algorithm, Relu functions are used as activation functions for the Actor primary network, and the activation function for the target network is the sigmoid function. The update rate for each network parameter is 0.01 and the learning rate for each network is set to 0.00001. The path formed by healthcare supply chain to achieve the maximum economic, social and environmental benefits under ideal conditions is set as the target mode. In this study, the setting of the target mode of the healthcare supply chain is shown in Figure 4.

In order to verify the effect of AI selection method, after analyzing a large number of literature and cases (52–54), the paper selects big data selection method, basic selection method and neural network selection method to compare with AI selection method, and presents the experimental comparison results through different curves.

### 4.2 Experimental results

**Figure 5:**
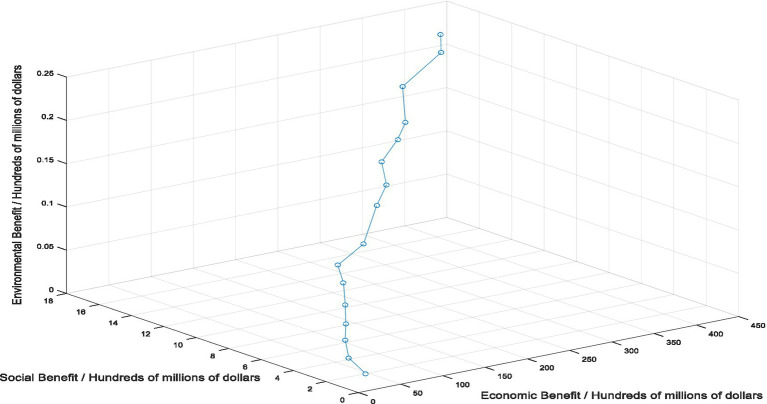
Healthcare supply chain mode selection results based on DDPG algorithm.

**Figure 6:**
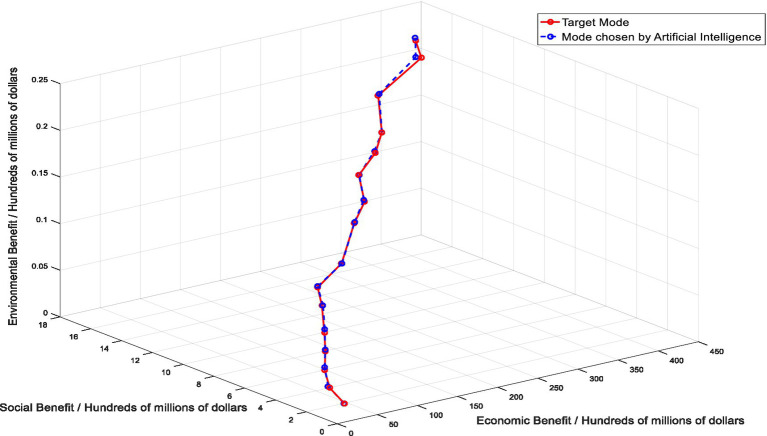
Comparison between target mode and AI-based mode.

DDPG algorithm generates actions through Actor network, and the collected data (state-action) is trained on the algorithm model. The results of healthcare supply chain mode selection based on DDPG algorithm can be obtained from the simulation data in Figure 5. From the experimental results, we can intuitively see the path of the healthcare supply chain mode selected by DDPG algorithm in the three dimensions of economy, society and environment. Whether it is in economic benefit dimension, social benefit dimension or environmental dimension, it is on the rise, and the highest economic benefit is $44.27 billion, the highest social benefit is $1.8 billion, the highest environmental benefit is $21.3 million. In order to verify the effect of application of artificial intelligence algorithm in healthcare supply chain mode selection, the results of healthcare supply chain mode selected based on DDPG algorithm were compared and analyzed with the results of target mode, as shown in Figure 6. Through comparative analysis, it can be seen that the results of healthcare supply chain mode selection obtained by artificial intelligence algorithm are consistent with the results of target mode. There are many overlapping points and the highest points of economic, social and environmental benefits almost coincide. Therefore, it can be seen that the application effect of artificial intelligence in healthcare supply chain mode selection is good, and artificial intelligence can help healthcare supply chain choose the optimal target mode.

**Figure 7:**
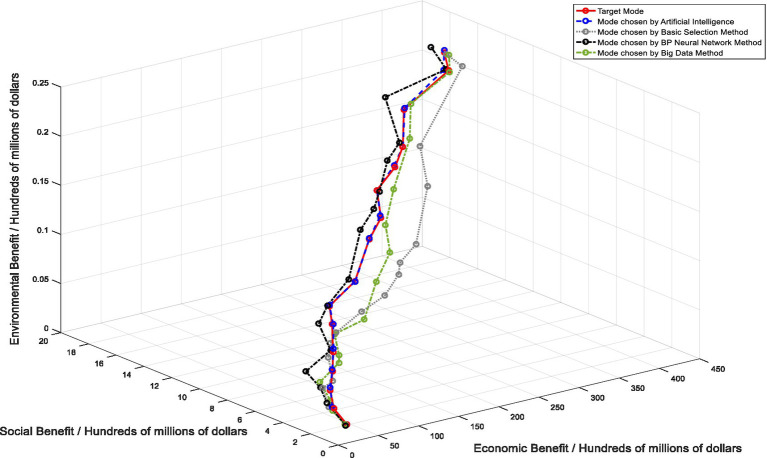
Comparison of target mode and multiple method selection results.

In order to conduct a more detailed analysis of the application effect of artificial intelligence, this study introduces three other commonly used methods for comparison. Under the same experimental environment, we conducted simulation experiments on healthcare supply chain mode selected by different methods, as shown in Figure 7. Different healthcare supply chain mode selection results can be clearly seen from the figure. First of all, in healthcare supply chain mode selection results obtained by the basic selection method, the highest economic benefit is $44.19 billion, the highest social benefit is $1.78 billion, and the highest environmental benefit is $21 million, which is far from the result of the target mode. It can be seen that the application of this method in healthcare supply chain mode selection still has certain defects. This method cannot accurately screen out the optimal mode of healthcare supply chain. Secondly, in the results of healthcare supply chain mode based on the neural network selection method, the highest economic benefit is $44.37 billion, the highest social benefit is $1.9 billion, and the highest environmental benefit is $21 million, which is still a certain gap with the target mode, although the highest point is slightly higher than the target mode and the middle part is relatively close to the target mode. However, on the whole, healthcare supply chain mode selected by this method is relatively ideal, and it is difficult to implement in combination with the reality and complex situation. Finally, according to the results of healthcare supply chain mode obtained by the big data selection method, the highest economic benefit is $44.192 billion, the highest social benefit is $1.76 billion, and the highest environmental benefit is $21 million, which is also inconsistent with the results of the target mode. Although there are overlapping parts, the whole path still deviates from the path of the target mode.

To sum up, healthcare supply chain mode selected by the above three methods has certain errors with the target mode, that is to say, the existing commonly used research methods cannot effectively select the optimal healthcare supply chain mode, and it needs to be improved. In addition, after comparing the results in this part, it can be found that healthcare supply chain mode selected by artificial intelligence algorithm is the closest to the target mode with the smallest error. Therefore, the simulation experiment in this part can verify that artificial intelligence is more effective in healthcare supply chain mode selection, compared with other methods. Artificial intelligence has more advantages in the choice of healthcare supply chain mode, which can ensure the quality of healthcare supply chain mode selection.

## 5 Discussion

According to the results of this study, as shown in Figure 5, the path presented by healthcare supply chain mode based on DDPG algorithm is basically consistent with the path presented by the target mode, and the error between the two is very small, which indicates the effectiveness of the application of artificial intelligence technology. In the choice of healthcare supply chain mode, artificial intelligence technology can help healthcare supply chain make intelligent decisions and choose the best operation mode. However, the application of artificial intelligence technology in the selection of healthcare supply chain mode is blank, which limits the practical application of artificial intelligence in the medical field. In fact, there have been some achievements in the application of artificial intelligence technology in the healthcare field, including the use of artificial intelligence technology for disease diagnosis and surgery (55, 56), and the use of artificial intelligence technology in these fields has been proven to be effective, and its accuracy even exceeds that of human medical experts. The development of artificial intelligence has undoubtedly brought revolutionary innovation to the medical industry, and the application of artificial intelligence algorithms in healthcare supply chain has also helped the medical industry to create a more intelligent and secure system, which is conducive to the improvement of the overall resilience level of healthcare supply chain, and is more conducive to the sustainable development of healthcare supply chain.

Therefore, this paper fills the gap in the application of artificial intelligence in the field of healthcare supply chain, and innovatively incorporates artificial intelligence technology into the choice of healthcare supply chain mode, which is not only conducive to the practical value of artificial intelligence technology, but also conducive to the improvement of the modern management level of healthcare supply chain. Specifically, firstly, healthcare supply chain model selection driven by artificial intelligence can better predict demand and manage inventory. AI algorithms can analyze historical patient data, disease trends, and other relevant factors to forecast the demand for medical supplies, pharmaceuticals, and equipment. Accurate demand forecasting can help healthcare organizations optimize inventory levels, reduce stockouts, and minimize excess inventory, leading to cost savings and improved operational efficiency. Secondly, the inclusion of artificial intelligence system in mode selection can provide decision support for managers. Artificial intelligence algorithm can analyze complex data sets and determine the most effective supply chain mode according to numerous indicators, help managers optimize the operation process of healthcare supply chain and improve the supply chain structure, so as to improve the efficiency of healthcare supply chain. Finally, the process of using AI for mode selection often involves supply chain risk management and resource allocation. Artificial intelligence can be used to identify risks in healthcare supply chain to ensure the continuity of supply chain operations and prevent supply chain interruptions. Artificial intelligence can also optimize resource allocation in healthcare supply chain and ensure the real-time supply of medical resources.

In addition, in order to further illustrate the advantages of artificial intelligence in healthcare supply chain mode selection and verify that artificial intelligence is superior to conventional methods, we selected three other commonly used methods for comparison. The first is the basic selection method, that is, to evaluate the key indicators of healthcare supply chain, and then select the corresponding mode according to the key indicator data of healthcare supply chain. This is the most common and commonly used selection method, but with the advent of the era of big data and the development of emerging technologies, this method has been gradually replaced by other methods. The second, neural network selection approach, is a computational model inspired by the structure and function of the human brain. It consists of interconnected nodes, or “neurons” that work together to process and analyze complex data. In a neural network, information is processed through layers of interconnected nodes, each of which applies a mathematical function to the input data and passes the results to the next layer. Neural network methods are popular for their ability to process large and complex data sets, learn from experience, and generalize patterns from input data to make predictions or decisions. They are used in a wide range of fields, including finance, healthcare, robotics, and many others (57). The third is the Big Data selection approach, which uses appropriate big data analysis methods and tools to effectively analyze large and complex data sets. This involves considering various factors such as the specific objectives of the analysis, the type and source of the data, scalability requirements, data processing tools, data storage and retrieval methods, and data security and privacy considerations. A big data selection approach is essential for organizations to gain valuable insights from the vast amount of available data and make informed decisions (58). As shown in Figure 6, we have drawn five paths, among which the path of the target mode is the reference object of this experiment. It can be intuitively seen from the figure that the path of healthcare supply chain mode selected by the basic selection method, BP neural network method and big data method is different from the path of the target mode to some extent. Healthcare supply chain mode obtained by the basic selection method has a slightly larger gap with the target model, while healthcare supply chain mode selected by the neural network method and big data method is close to the target mode, but there is still a certain gap compared with healthcare supply chain mode selected by artificial intelligence. Only the healthcare supply chain mode selected by artificial intelligence is more in line with the target mode. It can be judged that the accuracy rate of artificial intelligence application in healthcare supply chain mode selection is higher than that of existing common research methods. The use of artificial intelligence greatly improves the effectiveness of selection results, and artificial intelligence is the most effective and advantageous method.

## 6 Conclusion

The combination of artificial intelligence and healthcare field has become a hot spot at present, and the potential of artificial intelligence in the healthcare supply chain is the focus of existing research. However, few studies have explained the choice of healthcare supply chain mode, and the existing research methods are not systematic and effective. In order to make up for this shortcoming, the paper applies artificial intelligence to the choice of healthcare supply chain mode and realizes the intelligent choice of healthcare supply chain mode. Aiming at the intelligent choice of healthcare supply chain mode, firstly, this paper determines the indicators of evaluating healthcare supply chain mode, and defines the relevant calculation methods to provide support for effectively choosing the optimal healthcare supply chain mode. Secondly, combined with evaluation indicators, artificial intelligence technology is used to identify different healthcare supply chain modes, and the intelligent selection optimization method of healthcare supply chain modes is proposed based on deep reinforcement learning algorithm. Finally, the simulation experiment is constructed to carry out quality verification of the effect of artificial intelligence technology application, and the effectiveness of artificial intelligence application is demonstrated by comparing with different methods. The research results show that the path presented by the healthcare supply chain mode based on DDPG algorithm is basically consistent with the path presented by the target mode, which indicates the effectiveness of the application of artificial intelligence technology, and the path generated by the healthcare supply chain mode selected by the basic selection method, BP neural network method and big data method is different from the path of the target mode. This study not only makes a theoretical contribution, but also has practical significance. Further, we also point out the limitations of the study and the direction of future research.

### 6.1 Implications

In the current highly uncertain market environment, in order to promote the sustainable development of healthcare supply chain, this paper starts from the key point of healthcare supply chain mode selection, studies the intelligent choice of healthcare supply chain mode, applies artificial intelligence to the field of healthcare supply chain innovationally, and demonstrates the potential of artificial intelligence in the application of healthcare supply chain mode selection. The theoretical contribution of this study is to propose a more scalable and intelligent deep reinforcement learning algorithm to optimize the mode selection process of healthcare supply chain. By demonstrating the applicability of artificial intelligence in unknown environment and complex decision making, this algorithm demonstrates the rationality of artificial intelligence algorithm for intelligent decision making of supply chain mode. It makes up for the lack of objectivity of existing research methods, limited data analysis and inapplicability to dynamic environment, and enriches the existing research method system of healthcare supply chain. The healthcare supply chain can utilize the decision-making framework of deep reinforcement learning algorithm to optimize the choice of healthcare supply chain mode, which deepens the theoretical application of artificial intelligence in the healthcare supply chain and provides valuable insights for the integrated development of artificial intelligence and health economy. In addition, the practical significance of this study lies in the optimization of healthcare supply chain management, help medical enterprises to reduce costs while ensuring the quality and safety of drugs, and enable healthcare supply chain enterprises to better understand the shortcomings in the operation of the supply chain, so as to better improve the resilience of enterprises in the highly uncertain and highly risky market environment, and quickly respond to changes in consumer demand. In the end, gain competitive advantage in the market.

### 6.2 Limitations and future prospects

There are some limitations and deficiencies in this study. First, this study is limited by conditions, and the data collected in the simulation experiment is relatively limited, and the area involved is relatively narrow. Future studies can expand the scope of investigation and collect more samples. Second, the paper compares the artificial intelligence method with the basic selection method, BP neural network method and big data method. Although it has certain credibility, the existing other selection methods have not been fully considered. Therefore, in future research, the application of other models and methods can be considered to further improve the content of this part.

In summary, this paper combines artificial intelligence with healthcare supply chain, studies the intelligent selection process of healthcare supply chain mode, and proposes an intelligent selection optimization method of healthcare supply chain mode based on deep reinforcement learning algorithm. Finally, the simulation experiment verifies the effect of artificial intelligence technology in the application of healthcare supply chain mode selection, proving that the application of artificial intelligence in healthcare supply chain can also be extended to a wider range of research, providing innovative technical and method support for the future development of the health field.

## Data availability statement

The raw data supporting the conclusions of this article will be made available by the authors, without undue reservation.

## Author contributions

PL: Conceptualization, Writing – original draft. LL: Conceptualization, Investigation, Writing – original draft. QC: Investigation, Writing – review & editing. YC: Formal analysis, Writing – review & editing. CL: Formal analysis, Investigation, Writing – review & editing. XL: Formal analysis, Writing – review & editing.
